# Developing a consolidated research framework for clinical allied health professionals practising in the UK

**DOI:** 10.1186/s12913-020-05650-3

**Published:** 2020-09-11

**Authors:** Jennifer Harris, Kate Grafton, Jo Cooke

**Affiliations:** 1 Paediatric MSK/Orthopaedics, Chesterfield Royal Hospital NHS Foundation Trust, Calow, Chesterfield, Derbyshire S44 5BL UK; 2 Council for Allied Health Professions in Research (CAHPR) Strategy Committee, University of Lincoln, Brayford Pool, Lincoln, LN6 7TS UK; 3 School of Health and Social Care, University of Lincoln, Brayford Pool, Lincoln, LN6 7TS UK; 4 School for Health Science, University of Sheffield, Sheffield, S10 2TG UK

**Keywords:** Research competencies, Allied health professional, Research capacity, Applied research workforce

## Abstract

**Background:**

Allied Health Professionals (AHPs) form a significant part of the healthcare workforce and have great potential to improve services through research and research-informed practice. However, there is a lack of tradition in research embedded in practice in these professional groups. Barriers include clinical caseload pressures, a lack of sustainable training and consequent lack of confidence in practitioners. Practice managers are ill-equipped to monitor and guide staff research development. The modern healthcare system is a multi-disciplinary environment focused on the needs of the patient. A common framework across all AHP disciplines, offering equality in research knowledge and skills and shared language, might be helpful in planning and developing clinical career pathways. Our aim is to develop a consolidated research framework to help AHPs to plan and guide research activity throughout their career.

**Methods:**

The study was conducted in three phases. Phase one identified existing AHP research frameworks (AHPRF) through expert consultations and literature searches. Phase two involved framework analysis of the AHPRFs to develop a single consolidated framework. Phase three included a workshop with experts to validate and adapt the framework for practice.

**Results:**

Nineteen AHPRFs were identified. A consolidated framework was shaped by analysis of the AHPRFs resulting in a consolidated framework of eight sections, each containing a series of statements. Each section relates to an analytic theme within the framework analysis, and the statements were based on sub-categories of themes. The final framework was further shaped by the phase three workshop into a set of ‘stem’ statements that can be adapted to reflect different levels of expertise and the inclusion of a set of guiding principles developed through expert consultation.

**Conclusion:**

The consolidated framework was entitled ‘Shaping Better Practice Through Research: A Practitioner Framework’ by stakeholders, thus emphasising its ambition to embed research activity into practice. It instigates a new perspective within AHP research by offering practitioners and managers a tool that can be applied across public, private, and voluntary settings for AHPs in all disciplines. Its ambition is to develop capacity in the AHPs that can undertake research to improve services and the health of service users.

## Background

Allied Health Professionals (AHPs) constitute a large proportion of the international healthcare workforce offering great potential to increase the quality of patient and population health and to improve services through research [1]. AHPs make up approximately one third of the health and social care workforce in the UK with over 65,500 qualified staff registered with the NHS in 2018 [2]. The term ‘Allied Health Professionals’ is used within the UK to describe a diverse range of 14 autonomous professionals including physiotherapists, occupational therapists, radiographers, paramedics, speech and language therapists, podiatrists, dietitians, operating department practitioners, orthoptists, osteopaths, prosthetists and orthotists, art therapists, music therapists and dance therapists [3]. Although the scope of each of these professions is unique, they collectively offer holistic care within the domains of prevention, health promotion, diagnosis, treatment, support and enabling independence [4]. The breadth and range of skills and delivery of care within the public, private and voluntary sector offer AHPs unique opportunities to impact lives and transform the health and wellbeing of our changing population [5].

Evidence-informed practice is a core principle across all allied health disciplines and is a key component of pre-registration training [6–8]. Many initiatives support engagement, involvement and the delivery of evidence-informed practice, and skilled AHP researchers add impact and value to all levels of health and social care [9–12]. Health and social care organisations that engage in high quality and person-centred research activity have demonstrated higher rates of patient satisfaction, reduced mortality, improved quality performance, and improved organisational efficiency [12, 13]. At a departmental level, strong research culture is associated with reduced staff turnover and faster translation of evidence into practice with potential to improve patient outcomes, patient satisfaction and resource efficiency [12, 13]. However, when asked to consider why they choose to be involved in research, individual practitioners list personal interest in the topic, improved job satisfaction and career progression, recognition and professional kudos, increased awareness of research findings and the reward of seeing impact on practice amongst their reasons [9, 12, 13]. The National Institute for Health Research (NIHR) Clinical Research Network’s AHPs Strategy 2018–2020 [7] recognises that realising the research potential of AHPs is core to delivering the NIHR’s mission “to provide a health research system in which the NHS supports outstanding individuals, working in world class facilities, conducting leading edge research which is focused on the needs of patients and the public”. This reflects global health and social care policies [14–17].

Research capacity building is defined as “a process of individual and institutional development which leads to higher levels of skills and greater ability to perform useful research” ([18] , p. 1322). Building research capacity in frontline health and social care practitioners is essential to the development of a thriving research culture that offers value and meaning to patients and the public [19]. Within the context of allied health, the aim of this process is to “strengthen existing practitioner expertise with complementary research” ([19], p. 56) in order to enable high quality practice and advancement of the profession. Much effort has been made in recent decades to build research capacity and embed research cultures within the allied health professions [20–24]. Despite this, several barriers have been identified to establishing an effective research culture within this sector [1, 8].

A recent systematic review by Borkowski, et al. [8] highlighted a lack of confidence in research skills to be a major barrier to building a positive research culture amongst allied health professionals. Many AHPs perceive their knowledge and skills to be inferior, and opportunities for continued learning and development in research is considered lacking for practising clinicians [1, 8]. Practitioners describe high workload with limited time or resources to focus on research activity and sporadic support from managers [1, 8, 25]. The research literacy of individual managers within allied health is also varied, leaving many ill-equipped to support staff research development or signpost to experienced clinical academics [11, 26, 27]. This suggests further support is needed to enable all individual practitioners to continue to develop research skills, and for allied health leaders to track and support the research abilities of others.

Although many allied health disciplines provide education and guidance for continuing professional development, the breadth and depth of research knowledge and skills described within these is variable as is the language used to describe similar terms [28]. The field of clinical and applied research is an increasingly multi-disciplinary context in which the same standards, regulatory requirements, and responsibilities are applied regardless of professional background [29]. Potential convergence and divergence in guidance by individual professional bodies is likely to act as a further barrier to research activity and engagement, and could create challenges for recruitment of appropriately skilled and competent researchers [1, 8, 29]. Language used to refer to research within academic institutions can also be perceived as intimidating to AHPs applying research to their own practice [1]. This suggests a common framework, acceptable to AHPs practising in all applied health and social care systems and consolidating key research skills, knowledge and abilities across the professions would be helpful in supporting a strong AHP research culture.

The Council for Allied Health Professions Research (CAHPR) consists of a strategic committee, regional hubs in the UK representing 13 AHP member professions in the development of research capacity and capability in the UK [30]. Funded by proportionate subscription made by each professional body, CAHPR aims to.

“develop AHP research, strengthen evidence of the professions’ value and impact for enhancing service user and community care, and enable the professions to speak with one voice on research issues, thereby raising their profile and increasing their influence” [30].

The NIHR Collaboration for Leadership in Applied Health Research and Care (CLAHRC) is a UK based network of collaborative partnerships between health, public services and higher education [31]. NIHR CLAHRC Yorkshire and Humber aims to improve patient outcomes through applied health research, implement findings into practice, and increase research capacity and engagement in NHS organisations [32]. This project was developed through a secondment opportunity co-funded by the CAHPR Yorkshire and South Yorkshire regional hub and NIHR CLAHRC Yorkshire and Humber following recognition of local and national need.

## Methods

### Aim

To develop a consolidated research framework that supports allied health professionals practising in all public, private, and voluntary sectors of health and social care to help plan and guide research activity throughout their career.

### Design and objectives

**Fig. 1:**
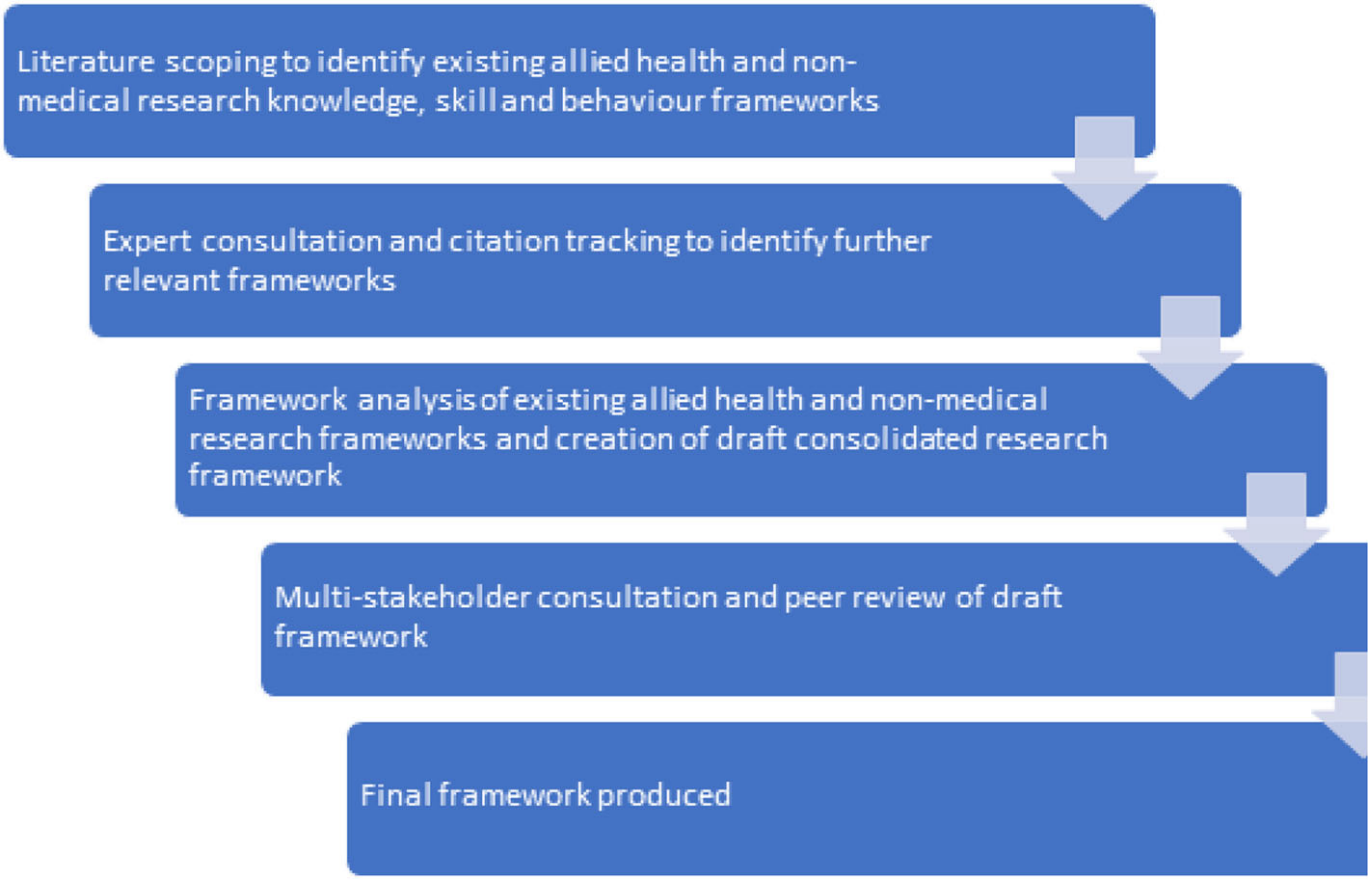
Study schematic

A three phase pragmatic approach was applied to develop this consolidated framework. Each phase aimed to achieve the objectives listed in Table 1. A schematic detailing study phases can be found in Fig. 1.

**Table 1:** Study phases and objectives

Study phase	Objective
Phase one	Identify existing AHP research frameworks (AHPRF) or research frameworks for other relevant non-medical health professions.
Phase two	Framework analysis of AHPRFs to produce one consolidated framework.
Phase three	Workshop of national experts to explore content and face validity of the consolidated framework, identify any relevant missing or superfluous components, consider practical application, and develop next steps.

### Phase one: identifying AHP research frameworks

This phase aimed to identify the scope and range of existing frameworks designed to support AHPs to develop research skill, knowledge, and behaviours.

Initial scoping of the literature commenced in February 2018 with a search of the Medline database (via Ovid) with search string detailed in supplementary materials. These findings were then supplemented with Google search using the term “clinical and academic skills, knowledge or behaviour in allied health and social care”. Expert consultation was also performed with key leaders within AHP research and professional development across the UK. These were identified through the websites of AHP professional bodies and with the authors and developers of AHPRFs identified through original literature searches and references and citations of key literature were examined for relevance [33]. Representatives from organisations listed in Table 2 were consulted in phase one to determine existing research frameworks specific to their discipline. JH, JC & KG considered relevance to the research question according to application of research knowledge, skills or behaviour in allied health or social care and non-medical health professions. For pragmatic reasons, phase one consultations were limited to those organisations for whom contact could be made within given timescales. However, saturation of statements was considered once significant overlap, duplication or lack of unique concepts, issues or themes was noted.

**Table 2:** : Organisations represented during initial consultation to identify AHPRFs

NIHR Clinical Research Network	The College of Podiatry
College of Paramedics	College of Occupational Therapists
Chartered Society of Physiotherapy	The British Association of Drama Therapists
Royal College of Speech & Language Therapists	The Royal Pharmaceutical Society
Society and College of Radiographers	Research and Development North West
British and Irish Orthoptic Society	

### Phase two: Framework analysis of AHPRFs to develop a consolidated framework

In phase two, the AHPRFs identified in the first phase underwent framework analysis to determine key themes applicable to research within allied health. Gale, et al’s 2013 [34] adaptation of Ritchie and Spencer’s Framework Method [35] offered the flexibility to compare and contrast data across numerous cases (AHPRFs) whilst maintaining clear steps and outputs.

After familiarisation with the AHPRFs, their contents were extracted and deductively coded using an analytical framework based on the Royal Pharmaceutical Society Research, Evidence Evaluation Toolkit [36]. Category headings taken from the REET framework were considered to offer ‘best fit’ in terms of systematic classification and comparison of coded data in a relatively simple format. These included research skills, methods and strategy, research knowledge, intellectual ability and personal qualities, research management and leadership, communication and dissemination of research, research education and training, working with others and collaborating in research, impact, evaluation and translation of research [36]. Early testing of this tool suggested clinician found this intuitive and easy to navigate [37]JH carried out initial coding and categorisation to develop a suitable analytical framework. JC independently coded approximately 20% of the data for data comparison and subsequent coding was agreed through consensus [36]. Codes were then classified into categories. JC and KG independently reviewed the data to identify additional patterns, consider outlying codes, and offer multiple perspectives as to relevance and repetition / duplication. Data were organised using Microsoft Excel software by JH. Emergent themes were identified during the analysis that further shaped the analytic framework.

JH, JC, and KG then met to convert the categorical data into statements organised under theme headings to create a new consolidated framework. The research team met to review language and terminology to ensure consistency throughout this new consolidated framework and ensure that themes and statements remained true to the original cases (AHPRFs). At the end of phase 2, a draft consolidated framework was produced ready for wider consultation with stakeholders.

### Phase three: consultation about content and next steps

The relevance and validity of the consolidated framework was established through multi-stakeholder consultation and peer review. The aims for the workshop were to share the draft framework with key representatives from AHP professional bodies and other relevant experts, to explore and validate the content domains within the framework, to identify missing or superfluous components,, to check suitable ‘entry level’ of each of statement from ‘awareness’ through to ‘advanced’ levels, to explore how AHPs might use the framework in practice, and to identify next steps for development.

A purposive sample of participants was mapped out to include representatives from AHP professional bodies, applied research capacity-building leads, clinicians, and managers from a range of organisations, and CAHPR strategy group members. Participants were approached via CAHPR strategy group and level of expertise was established via nomination through AHP professional bodies. Authors of some existing AHPRFs including the national NIHR workforce group were also participants.

The workshop also aimed to consider practical application and next steps in development. Following electronic distribution of draft consolidated framework, participants attended a face-to-face workshop facilitated by JH, JC & KG. Following introduction and overview of the project, participants were separated into three pre-determined sub-groups. Sub-group members were selected to offer maximum diversity in professional, research and practice backgrounds. Each group reviewed 2–3 themes of the consolidated framework, of approximately equal size, to consider accuracy of statements, clarity of description, missing or superfluous statements. Comments were recorded on flip chart paper. Larger group discussions offered opportunity for participants to share knowledge and expertise on research abilities within allied health and to make recommendations on how, and in what format, the consolidated framework could be used in practice. Recommended changes to framework content and layout were made through participant feedback and agreement in open committee. Potential for over-dominance by individuals or coalitions and reluctance to challenge long-held beliefs was limited through use of the Padlet collaborative interactive tool [38, 39]. This virtual graffiti wall allowed anonymous commentary from workshop participants and from those unable to attend in person encouraging greater collaboration and engagement from all workshop participants and offering instant visual feedback and review of key concepts and easy data collection [40].

Feedback and recommendations from this workshop were collected in written format on draft versions of the consolidated framework and digital format on the Padlet. Each member of the study team (JH, JC, and KG) was responsible for updating specific components of the framework, which were then collated and agreed by consensus to shape the final consolidated framework.

## Results

### Phase one: the AHPRFs identified

A total of 19 profession-specific and generic health and social care AHPRFs were identified in phase one. Original searches retrieved 45 studies but were excluded as they did not contain specific statements of knowledge, skill or behaviour. Details of the search string are found in the supplementary information (file 2). Google searches retrieved 11 studies and a further 8 were identified and retrieved through expert consultation. These reflected the breadth and diversity of applied research knowledge, skills, and behaviours relevant to AHPs in a variety health and social care settings and contexts. Please see Table 3 for details of the AHPRFs.

**Table 3:** AHP Research Frameworks identified in Phase one of project

RCN Competency Framework for Clinical Research Nurses [39]	RCN Competency Working Group	2011	Royal College of Nursing	Nurses	UK
Harmonized Core Competency Framework Vs. 2 [38]	Joint Task Force for Clinical Competency	2017	Joint Task Force for Clinical Competency	Clinical Research Professionals	Global
Vitae Researcher Development Framework [41]	Vitae	2010	Vitae Careers Research and Advisory Centre (CRAC) Limited	Researchers in Higher Education	UK
Clinical Academic Careers Pathway Capability Framework [42]	Westwood, G & Richardson, A	2012	The Association of UK University Hospitals	Nurses, Midwives and Allied Health Professionals	UK
NHS National job profile: Allied Health Professionals (Clinical Researcher) [43]	NHS Employers	2008	National Health Service	Allied Health Professionals	UK
Health Services & Policy Research Enriched Core Competencies [44]	Canadian Health Services and Policy Research Alliance Working Group	2017	Canadian Institutes of Health Research (CIHR)	Health services and policy research doctoral graduates	Canada
SPOR Capacity development framework [45]	SPOR External Advisory Committee on Training and Career Development	2015	Canadian Institute of Health Research	Patient-orientated clinical and health researchers	Canada
CSP Physiotherapy Framework [46]	CSP	2011	Chartered Society of Physiotherapy (CSP)	Physiotherapists	UK
Advanced practice in physiotherapy [47]	CSP	2016	Chartered Society of Physiotherapy (CSP)	Physiotherapy Advanced Practitioners	UK
RPS Research Evidence and Evaluation Toolkit (REET) [35]	RPS	2017	Royal Pharmaceutical Society (RPS)	Foundation and advanced pharmacists	UK
Dietitians and Research: A Knowledge and Skills Framework [48]	The British Dietetic Association Research Committee	2015	The British Dietetic Association (BDA)	Dietitians	UK
Speech & Language Therapists working in Consultant Roles [49]	RCSLT	2010	Royal College of Speech and Language Therapists (RCSLT)	Speech and Language Therapists	UK
Education and Career Framework for the Radiography Workforce [50]	Coleman, L	2013	The Society and College of Radiographers	Radiography workforce	UK
Career Framework Guide: Prosthetics & Orthotics [51]	Nicol, A	2013	The British Association of Prosthetists and Orthotists (BAPO)	Prosthetists and Orthotists	UK
Post Registration – Paramedic Career Framework [52]	The College of Paramedics	2018	The College of Paramedics	Paramedics	UK
Career Development Framework: Guiding Principles for Occupational Therapists [53]	RCOT	2017	The Royal College of Occupational Therapists (RCOT)	Occupational Therapists	UK
East Sussex Research Escalator Tool© [18]	Canby, A, McCrum, C & Poole, K	2017	East Sussex Healthcare NHS Trust, NHS England, Council for Allied Health Professions in Research (CAHPR)	Health professionals	UK
RESearch Self-Assessment Tool (RESSAT) [19]	Grafton, K	2017	Sheffield Hallam University	AHPs in Higher Education	UK
^a^*Orthoptics Curriculum Framework* [54]	*Horwood, A*	*2016*	*The British & Irish Orthoptic Society*	*Orthoptists*	UK & ROI

^a^Included within phase three iteration

### Phase two: themes and subcategories identified to shape the draft framework

Eight broad themes of AHP practitioner research knowledge, behaviour and skill were identified in phase two. Themes and sub-categories can be found in Table 4 below:

Themes were adapted from the REET framework [36] and shaped through the analysis of the data. Emergent themes included ‘research delivery’, and ‘career development’. The delivery theme was pulled out as it is highly relevant to current policy in the UK (see NIHR CRN Allied Health Professionals Strategy 2018–2020 [55]). Such data was evident in some subcategories of the AHPRFs, but not made visible as indicated in the current policy context, so we organised this data as an emergent theme. The latter was originally based on the ‘research knowledge, intellectual ability and personal qualities’ but include broader concepts of personal and career development.

A review of how the themes mapped against the original AHPRF can be seen in Table 5.

**Table 4:** Themes and sub-categories

	Theme	Theme description *Includes knowledge skills and behaviour*	Subcategories
1.	Research methodology and methods	Different epistemologies, approaches and techniques used to develop research and gain research evidence. Includes applying the appropriate approaches to address research questions being asked	Scientific concepts and application of research knowledge
			Analysis *(process of looking for patterns in information, either quantitative or qualitative)*
			Proposal development
2.	Research strategy and planning	Management of projects in an ethical manner in a dynamic health and social care environment. Meeting deadlines. Forward planning to maximise capacity and impact	Applied research strategy and policy
			Research project planning and development
3.	Research delivery	Execution of research safely and effectively across a range of contexts	Ethics, Safety and informed consent
			Operation of research
4.	Research management and leadership	Leading and managing research activity	Leadership and management in research
			Management and leadership in projects
5.	Research education and training	Education and training of the wider workforce in research and EBP including clinical and/or research supervision and mentoring others	Education General (any setting)
			Clinical Education
			Academic Education
6.	Working with others and collaborating in research	Developing effective, trusting research relationships with other researchers, patients, professionals, policy makers and others colleagues to build and sustain a collaborative research activity	Networking
7.	Research-informed practice, dissemination, and impact	Use of evidence to develop and inform practice, dissemination, and knowledge mobilisation activities.	Translation of knowledge into practice
			Dissemination of own research
			Impactful Activities
8.	Own career development	Planning steps in personal growth and career.	Career development knowledge and skills

**Table 5:** Content of existing AHPRFs and how they map against consolidated framework categories

Consolidated Framework themes		Integrated workforce framework [45]	RCN Competency Framework for Clinical Research Nurses [42]	Harmonized Core Competency Framework [43]	Vitae Researcher Development Framework [55]	Clinical Academic Careers Pathway Capability Framework [56]	NHS National job profile: Allied Health Professionals (Clinical Researcher) [57]	Health Services & Policy Research Enriched Core Competencies [51]	SPOR Capacity development framework [52]	CSP Physiotherapy Framework [53]	Advanced practice in physiotherapy [54]	RPS Research Evidence and Evaluation Toolkit (REET) [36]	Dietitians and Research: A Knowledge and Skills Framework [60]	Speech & Language Therapists working in Consultant Roles [61]	Education and Career Framework for the Radiography Workforce [62]	Career Framework Guide: Prosthetics & Orthotics [63]	Post Registration – Paramedic Career Framework [64]	Career Development Framework: Guiding Principles for Occupational Therapy [65]	East Sussex Research Escalator Tool [66]	RES earch Self-Assessment Tool (RESSAT) [67]
1.	*Research methodology & methods*	✓	✓	✓	✓	✓	✓	✓		✓	✓	✓	✓	✓	✓	✓	✓	✓	✓	✓
2.	*Research strategy & planning*	✓	✓	✓	✓	✓	✓	✓	✓	✓	✓	✓	✓	✓	✓	✓	✓	✓	✓	✓
3.	*Research delivery*	✓	✓	✓	✓	✓	✓	✓	✓	✓	✓		✓	✓	✓	✓	✓	✓	✓	✓
4.	*Research management & leadership*	✓	✓	✓	✓	✓	✓	✓		✓	✓	✓	✓	✓	✓	✓	✓	✓	✓	✓
5.	*Research education & training*		✓	✓	✓	✓	✓					✓	✓	✓	✓	✓	✓	✓	✓	✓
6.	*Working with others & collaborating in research*	✓	✓	✓	✓	✓	✓	✓	✓			✓	✓	✓	✓	✓		✓	✓	✓
7.	*Research-informed Practice, Dissemination and Impact*	✓	✓	✓	✓	✓	✓	✓	✓	✓	✓	✓	✓	✓	✓	✓	✓	✓	✓	✓
8.	*Own career development*				✓	✓	✓			✓	✓									

It can be seen that the area that is not included in most of the original AHPRFs is that of career development and planning. Gaps in the education and planning were also evident in many. Three [56–58] of the original frameworks did include some content within all of the themes of the consolidated framework, but they did not include the full range of subcategories identified through the analysis of all of the documents. A few additional elements were included as a result of expert opinion from the workshop. This included developing skills in co-production of research with stakeholders, and supporting outputs from research that are directly useful for practice, which the CLAHRC defines as ‘actionable outputs’ [41, 44, 45]. Any recommendations considered not within the scope of initial development were factored into the second stage of project development. Thus, the consolidated framework helped to include a full and comprehensive addition to the existing AHPRFs.

The data analysis revealed subcategories within each of the themes listed above. A series of statements were developed to reflect each subcategory, thereby generating the detail of the draft-consolidated framework. In doing this we reflected that the abilities identified operated at a range of expertise, from research awareness needed for all practitioners to an advanced level for research leaders. Before going out to consultation, members of the research team (JC, KG) used Integrated Workforce Framework levels of awareness, core, intermediate and advanced levels [43] to allocate the level of performance the statement reflected [27]. These allocated levels were used to encourage discussion at the phase three workshop. A selected example of the draft framework that went out to consultation is given in Table 6.

**Table 6:** Example of draft framework

B. Research Project Planning and Development	
**Knowledge**	
Has knowledge of a range of study designs and methodologies relevant to clinical research	Core
Understanding of different phases of research process	Core
Knowledge of the requirements for Public involvement in research	Core
Awareness of regulatory and legal frameworks and implications for clinical research design and development	Intermediate
Understanding of funding sources	Intermediate
Understanding of financial management in the design and conduct of research	Intermediate
**Skills and behaviour**	
Identifies problems and issues arising from practice and develops research questions based on this	Awareness
Develops research teams appropriate to the research methods	Intermediate
Writes research proposals	Intermediate
Successfully applies for grants and fellowships	Intermediate
Designs research studies using appropriate method for the research question	Intermediate
Describes and summarise specific processes essential to ensure regulatory approval	Intermediate
Adjusts design appropriately when unforeseen problems arise	Intermediate
Plans and coordinates detailed research programmes	Advanced

### Phase three: findings from the workshop regarding content and next steps

Twelve participants attended the workshop, and a further two participants provided written comments on the draft-consolidated framework as they were unable to attend. Invited participants included people with wide range of experience and expertise including four members of the CAPHR strategy group, three representatives from NIHR Clinical Research Network (CRN), two regional research training providers, and three clinicians who were both research and clinically active. Two national workforce planning policy representatives also attended. Most of the group were AHP trained including three radiographers, a speech and language therapist, three physiotherapists and a dietician, an orthoptist, and an occupational therapist.

Workshop participants reviewed each theme of the consolidated framework. Statements were adjusted to ensure consistency in language, clarity, and suitability across the range of practice settings and AHP roles. It was highlighted that many AHPs work across the health and social care system, and that some work in private practice. The final framework needed to embrace this, and so participants advised that the terminology moved away from clinical research language and be replaced with the term ‘applied research’ that reflected its application in different contexts.

The title was also changed from ‘Clinical Research Skills and Knowledge Framework’ to ‘Shaping Better Practice through Research: A Practitioner Framework’ to reflect a practitioner and practice focus.

**Fig. 2:**
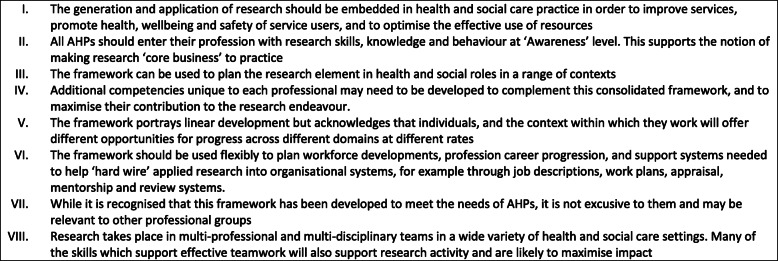
Guiding principles to set the context of using the consolidated framework

A small number of additional statements were incorporated following recommendation and agreement of participants in consultation workshop. These included an expansion of competencies around public and patient involvement, and a stronger emphasis of working with wider stakeholders. Developing and influencing research capacity was thought to be an important element of research leadership. An increased focus on research-informed teaching in clinical practice was also expanded upon. Some statements were re-categorised. For example, statements related to grant and fellowship were moved from the ‘research strategy and planning’ section to ‘research methodology and methods’. Other skills were incorporated within overarching principles as they were considered pertinent to all research activities across the consolidated framework, for example team-working skills were incorporated into overarching principles (see Fig. 2 VII and VIII).

Workshop participants made recommendations regarding presentation of the consolidated framework including techniques to make the framework easier to navigate and increase usability such as visual icons to represent each theme, use of a glossary, consistency in terminology and use of case examples as appendices.

Discussions ensued about expertise level. As a result of this, statements were developed to a series of ‘stem statements’ where the important aspect of each statement was highlighted in bold. The term ‘stem’ statement reflects a term used to describe “a central part of something from which other parts can develop or grow, or which forms a support” [59]. It is anticipated that these stem statements will stimulate additions to our framework reflecting differing levels of expertise but linked to the original concepts in the consolidated framework. The entry level could be considered the start of a spectrum of abilities linked to the stem statement. In practice subsequent levels will build on the entry-level statement. An example of how a stem statement can be developed to reflect increase in expertise is given in Table 7.

**Table 7:** Stem statements

Stem statement: Research, audit and service evaluation	
Awareness	Able to differentiate between research, audit and service evaluation
Core	Able to plan and deliver audit and contribute to service evaluation projects
Intermediate	Able to plan and deliver audit, service evaluation and research projects
Advanced	Uses service evaluations to promote service change and prepare for research grant proposals

There was some debate about the entry level for each statement and changes made. Details of level of expertise can be found in Table 8. It was agreed that the entry level for some stem statements would start at the higher level of expertise. For example, statements relating to research leadership, the process of applying for research grants and external funding, and co-ordination of research programmes may only become relevant at ‘core’ or ‘intermediate’ levels of expertise. Allied health professionals working at ‘awareness’ level of expertise would not be expected to demonstrate knowledge, skill or behaviour in every sub-domain. The final framework includes stem statements with a suggested entry level, but these are only tentative, and more work is needed here to establish consensus. A section of the resultant consolidated framework is given in Table 9. Further details can be found in supplementary information (file 1).

**Table 8:** A description of levels of expertise (adapted from NIHR / CRN Integrated Workforce Framework (IWF) levels [44])

***Awareness***	***Awareness of the applied research context and who/where to go to if xyz happens. Demonstrate understanding of how your work fits within this context.***	e.g. ***Junior Practitioner***
***Core***	***Have working knowledge and skill within your working area.*** **i.e.** ***not assumed to be transferrable; can be learnt even if technically tricky where the context is predictable. Able to support Awareness level. Work under guidance and within defined parameters and make judgements between a predefined range of options.***	**e.g.** ***Established Practitioner***
***Intermediate***	***Able to transfer/adapt knowledge and skill to different areas/topics that may be unpredictable. Able to support the Core and Awareness levels. Prioritises own work/activities, demonstrates experience of working in a complex environment and shows creativity in developing solutions by determining the options.***	**e.g.** ***Clinical Researcher*** ***Advanced Clinical Practitioner*** ***Advanced Specialist Practitioners***
***Advanced***	***Able to apply knowledge and skill in highly complex and unpredictable research areas and contexts. Able to support all other levels. Provides leadership and takes overall responsibility, making complex or highly complex judgements. Conceives, designs develops and adapts solutions through critical analysis, evaluation and synthesis.***	***Advanced Specialist Practitioners*** ***Consultant Practitioner*** ***Professor of Clinical Research/ Practice***

**Table 9:** A selected example of the consolidated framework

Research Methodology and Methods	
**A. Scientific concepts and application of research knowledge**	**Entry Level**
Broad awareness of **knowledge creation processes**	Awareness
Awareness of basic **theoretical concepts** and **methodologies** in relation to applied research	Awareness
Able to differentiate between **research, audit and service evaluation**	Awareness
Applies **technical language** with applied research e.g. research participant compared to patient data compare to information statistical significance compared to clinical significance	Awareness
Selects **appropriate research methods to answer research questions**	Awareness
Critiques and selects appropriate **outcome measures / tools** in research projects	Awareness
Develops **research questions** by considering research area and ‘real-world’ affairs	Core
**Application of theoretical concepts and methodologies** in relation to clinical research	Intermediate
Awareness of relevant research **methodological developments in field of interest**	Intermediate
**Uses multiple sources of evidence** (including stakeholder and user involvement / co-production) in research development	Intermediate
Articulates own **assumptions** and constructs and sustains arguments in a clear, evidenced and concise manner	Intermediate
Work with stakeholders throughout the research process	Intermediate
**B. Analysis**	**Entry Level**
Is aware of appropriate tools and systems in the **search for evidence** e.g. databases	Awareness
**Information Technology** (IT) literate For example, use of Excel, word	Awareness
Understands how to **interpret** qualitative and quantitative research data	Awareness
Undertakes appropriate **data analysis**	Core
Uses appropriate **tools** to collect data and measure outcomes	Core
**C. Proposal development**	**Entry Level**
Applies for **funding** grants and fellowships	Intermediate
**Designs research studies** using appropriate method for the research question	Intermediate
Writes research proposals that **adhere to requirements** of funding bodies, ethics and governance processes	Intermediate
Plans and leads detailed **research programmes**	Advanced

### How the consolidated framework should be used: principles for application

Participants considered that the consolidated framework should be implemented flexibly to inform conversations about research skills and career development with practitioners, managers, and policy-makers. It was advised that the consolidated framework should not be used as a linear model to map performance objectives or pay, but should inform discussions for career planning, and support integrating research activity into everyday practice. It could be incorporated into, or used alongside existing appraisal systems, and in local and national workforce planning, policies and guidance. The ambition would be to develop a space for discussion and reflection, to help plan a future practice- based workforce that conducts and delivers research within their practice.

As a result of the phase three workshop, ‘Research Practitioner Framework Guiding Principles’ were developed reflecting the workshop discussions. These are given in Fig. 2.

A further AHPRF was identified during in the workshop [54]. All concepts within the relevant research and literature skills section of this AHPRF were reviewed by JC and JH and considered present in the consolidated framework, implying a saturation of the data.

The final framework was shared with members of the CAHPR strategy group who approved all changes. The full framework is available on the CAHPR website at https://cahpr.csp.org.uk/content/cahpr-research-practitioner-framework

## Discussion

‘Shaping Better Practice Through Research: A Practitioner Framework’ offers a consolidated framework that sets out the knowledge and skills recommended for AHPs to carry out a variety of research activities within their practice, with potential to enhance and support AHP research capacity and culture. It offers a new perspective within AHP research by offering practitioners and managers a tool that can be applied across public, private and voluntary settings for AHPs in all disciplines.

Although there were many similarities across the AHPRFs analysed within this project, ‘delivery of applied research’ was a useful emergent theme that offered guidance on specific competencies required by AHPs engaging in, and delivering research in practice settings. It reflects an important development in the UK in the role of the NIHR CRN which supports centrally funded research delivery across the whole of the NHS by practitioners including AHPs. Stem statements within this category offer consistency in expectations across AHPs but also reflect knowledge, skills and behaviours identified as critical in research delivery across fields of medicine, nursing and other non-medical professions such as pharmacy [36, 42, 46]. Opportunities to develop competence and confidence in the operations of research delivery will promote safety, ethics and legal regulations to build research capacity [24] and reflect international regulation [47].

A further emergent theme within the consolidated framework was ‘own career development’. Over recent decades, a number of specialised allied health roles have developed in response to changing health and social policies. These reflect a shift in focus on restoring and maintaining financial balance and delivering core quality standards that are fit to accommodate the needs of an aging population [5, 48, 49]. This has included flexibility in role boundaries, extended scope or advanced clinical practice, and emergence of allied health research positions [10, 50]. Although individual career progression within allied health is likely to be informed by profession-specific requirements and health and social care policy, engagement in research is considered the most over-looked of the four pillars of advanced practice [51]. Frameworks such as the Vitae Researcher Development Framework [56] have been traditionally used within academic settings in the UK to map research career development but is not commonly implemented in practice-based environments. This is a useful extension to the consolidated framework AHP clinicians considering an academic or clinical academic research career and may facilitate discussions across sectors enabling joint appointments and other new career pathways.

The consolidated framework ensures knowledge, skills and behaviours associated with individual AHP research practice reflects national and international policy and regulation. The inclusion of internationally recognised research frameworks [42, 52, 53], and to national job profiles [43, 58] as well as expertise gained from phase three of this project will facilitate workforce planning across practice settings. This combination of concepts and review by a multi-stakeholder audience can promote a shared research language and offer collaboration across teams, services and organisations including universities and industries [24, 25]. As reflected in the guiding principles, this framework aims to support individual practitioners, managers, academics, and policy makers to facilitate and plan research activities using a shared language.

### Future developments

The current iteration of ‘Shaping Better Practice Through Research: A Practitioner Framework’, offers stem-statements under eight theme headings that can be used by the range of AHPs. It is acknowledged that the level and rate at which a practitioner will be expected to advance through each category will vary according to the specific AHP role, opportunities, and service need.

In common with international frameworks, future developments of the consolidated framework are likely to benefit from statements that identify both “what to do” and “how to do it” [29]. Although early iterations of the consolidated framework mapped statement levels in line with NIHR / CRN Integrated Workforce Framework (IWF) levels [43], it was not within the scope of this project to gain consensus on levels of progression that reflected all professional roles and practice settings and international qualifications frameworks, nor was it possible to determine if registered AHPs in the UK are entering the profession with research skills, knowledge and behaviours at an ‘Awareness’ level. This is a limitation of the findings. Additionally, phase three of this project recognised further iterations of the consolidated framework should include case exemplars mapping elements of the framework against research-specific roles in a range of contexts. As recognised by our expert panel and mentioned within guiding principles, effective implementation of this framework will rely on appropriate support systems and has potential to impact pre- and post-registration training, job descriptions and workforce planning.

Future iterations of ‘Shaping Better Practice Through Research: A Practitioner Framework’ are likely to require consensus through Delphi study including input from international AHP representatives and further consultation and piloting in practice-based environments.

### Limitations

This project was completed with time and resource constraints and, therefore, followed a pragmatic approach that reflected the funding available. The data that informed the consolidated framework reflects the analysis of existing framework with expert opinion and experience. The wider literature was not used and not all 15 AHP disciplines supplied AHPRFs. This is a limitation. Original AHPRFs were predominantly based in a UK setting limiting transferability across geographical and political settings. We were also unable to establish consensus on the entry level of each stem statement due to time constraints, and this requires further work.

## Conclusions

‘Shaping Better Practice Through Research: A Practitioner Framework’ offers a consolidation of existing AHP research frameworks developed through framework analysis and expert consultation. This consolidated framework has the potential to support AHPs to fulfil their research potential by facilitating research-informed practice, research career and activity planning across a variety of practice-settings. By offering a coordinated approach and shared language, this framework provides a unique opportunity to build research capacity in the allied health workforce and work together across health and social care systems to plan clinical academic careers, and to improve services and health of service users.

## Supplementary information

**Additional file 1.** Draft 1 Shaping Better Practice through Research: A Practitioner Framework.

**Additional file 2.** Search strategy Ovid (Medline).

## Data Availability

The datasets used and/or analysed during the current study are available from the corresponding author on reasonable request.

## Abbreviations

AHP: Allied Health Professional
AHPRF: Allied Health Profession Research Framework
BDA: The British Dietetic Association
CAHPR: Council for Allied Health Professions in Research
CRN: Clinical Research Network
CSP: Chartered Society of Physiotherapists
NHS: National Health Service
NIHR: National Institute for Health Research
NIHR CLAHRC YH: National Institute for Health Research Collaboration for Leadership in Applied Health Research & Care Yorkshire & Humber
NMAHPs: Nurses, midwives and allied health professionals
RCN: Royal College of Nursing
REET: Research Evidence Evaluation Toolkit
RESSAT: RESearch Self-Assessment Tool
RPS: Royal Pharmaceutical Society
RCSLT: Royal College of Speech and Language Therapists
SPOR: Strategy for Patient-Oriented Research
UK: United Kingdom

## References

[1] Pager S, Holden L, Golenko X (2012). Motivators, enablers, and barriers to building allied health research capacity. J Multidiscip Healthc.

[2] NHS Digital (2018). NHS workforce statistics: may 2018. In. England.

[3] Allied Health Professions [https://www.england.nhs.uk/ahp/] Accessed 20 May 2019.

[4] Dorning H, Bardsley M (2014). Focus on: allied health professionals can we measure quality of care?.

[5] Rastrick S (2017). Allied health professions into action: using allied health professions to transform health, care and wellbeing. In.: NHS England.

[6] Asokan GV (2012). Evidence-based practice curriculum in allied health professions for teaching-research-practice nexus. J Evidence-Based Med.

[7] Forrest JL, Miller SA (2001). Integrating evidence-based decision making into allied health curricula. J Allied Health.

[8] Borkowski D, McKinstry C, Cotchett M, Williams C, Haines T (2016). Research culture in allied health: a systematic review. Australian J Primary Health.

[9] Dimova S, Prideaux R, Ball S, Hashfield A, Carpenter A, Marjanovic S (2018). Enabling NHS staff to contribute to research: Reflecting on current practice and informing future opportunities.

[10] Wenke R, Mickan S (2016). The role and impact of research positions within health care settings in allied health: a systematic review. BMC Health Serv Res.

[11] Wenke RJ, Ward EC, Hickman I, Hulcombe J, Phillips R, Mickan S (2017). Allied health research positions: a qualitative evaluation of their impact. Health Res Policy Syst.

[12] Boaz A, Hanney S, Jones T, Soper B (2015). Does the engagement of clinicians and organisations in research improve healthcare performance: a three-stage review. BMJ Open.

[13] Harding K, Lynch L, Porter J, Taylor NF (2017). Organisational benefits of a strong research culture in a health service: a systematic review. Australian Health Rev.

[14] Department of Health & Social Care (2015). The NHS Consitution for England.

[15] Department of Health & Social Care (2017). Department of Health and Social Care Single Department Plan.

[16] Dixit SK, Sambasivan M (2018). A review of the Australian healthcare system: a policy perspective. SAGE Open Med.

[17] Tulloch-Reid MK, Saravia NG, Dennis RJ, Jaramillo A, Cuervo LG, Walker SP, Salicrup LA (2018). Strengthening institutional capacity for equitable health research: lessons from Latin America and the Caribbean. BMJ.

[18] Trostle J (1992). Research capacity building in international health: definitions, evaluations and strategies for success. Social Sci Med (1982).

[19] Pickstone C, Nancarrow S, Cooke J, Vernon W, Mountain G, Boyce RA, Campbell J (2008). Building research capacity in the allied health professions. Evidence Policy.

[20] Cooke J (2005). A framework to evaluate research capacity building in health care. BMC Fam Pract.

[21] Holden L, Pager S, Golenko X, Ware RS (2012). Validation of the research capacity and culture (RCC) tool: measuring RCC at individual, team and organisation levels. Aust J Prim Health.

[22] Slade SC, Philip K, Morris ME (2018). Frameworks for embedding a research culture in allied health practice: a rapid review. Health Res Policy Syst.

[23] Strickland K (2017). Developing an infrastructure to support clinical academic careers. Br J Nurs.

[24] Matus J, Walker A, Mickan S (2018). Research capacity building frameworks for allied health professionals – a systematic review. BMC Health Serv Res.

[25] Gifford WA, Squires JE, Angus DE, Ashley LA, Brosseau L, Craik JM, Domecq M-C, Egan M, Holyoke P, Juergensen L (2018). Managerial leadership for research use in nursing and allied health care professions: a systematic review. Implement Sci.

[26] Williams C, Miyazaki K, Borkowski D, McKinstry C, Cotchet M, Haines T (2015). Research capacity and culture of the Victorian public health allied health workforce is influenced by key research support staff and location. Australian Health Rev.

[27] Pain T, Plummer D, Pighills A, Harvey D (2015). Comparison of research experience and support needs of rural versus regional allied health professionals. Australian J Rural Health.

[28] Glasziou P, Burls A, Gilbert R (2008). Evidence based medicine and the medical curriculum. Bmj.

[29] Sonstein S, Seltzer J, Li R, Silva H, Jones C, Daemen E (2014). Moving from compliance to competency: A harmonized core competency framework for the clinical research professional, vol. 28.

[30] Council for Allied Health Professions Research: About CAHPR [https://cahpr.csp.org.uk/about-cahpr] Accessed 20 May 2019.

[31] NIHR Collaborations for Leadership in Applied Health Research and Care (CLAHRCs) [https://www.nihr.ac.uk/about-us/how-we-are-managed/our-structure/infrastructure/collaborations-for-leadership-in-applied-health-research-and-care.htm] Accessed 20 May 2019. 10.1186/s13012-018-0805-y PMC6094566 30111339

[32] NIHR CLAHRC Yorkshire and Humber [http://clahrc-yh.nihr.ac.uk/] Accessed 20 May 2019.

[33] Centre for Reviews and Dissemination (2009). Systematic Reviews; CRD’s guidance for undertaking reviews in health care.

[34] Gale NK, Heath G, Cameron E, Rashid S, Redwood S (2013). Using the framework method for the analysis of qualitative data in multi-disciplinary health research. BMC Med Res Methodol.

[35] Ritchie J, Lewis J (2003). Qualitative research practice: a guide for social science students and researchers.

[36] Research and evaluation toolkit (REET) [https://www.rpharms.com/resources/toolkits/research-evidence-and-evaluation-toolkit] Accessed 20 May 2019.

[37] The Royal Pharmaceutical Society (2017). RPS REET phase I testing. In: The Royal Pharmaceutical Society.

[38] Padlet; Collaborate better. Be more productive [https://en-gb.padlet.com/] Accessed 20 May 2019.

[39] Jones J, Hunter D (1995). Consensus methods for medical and health services research. Bmj.

[40] Fuchs B. The Writing is on the Wall: Using Padlet for Whole-Class Engagement, vol. 240: Library Faculty and Staff Publications; 2014.

[41] Cooke J, Langley J, Wolstenholme D, Hampshaw S (2017). **“**Seeing” the difference: the importance of visibility and action as a mark of “authenticity” in co-production: comment on “collaboration and co-production of knowledge in healthcare: opportunities and challenges”. Int J Health Policy Manag.

[42] Joint Task Force for Clinical Competency (2017). Harmonized Core Competency Framework for the Clinical Research Professional.

[43] Integrated Workforce Framework Copyright © 2017 NIHR CRN [https://sites.google.com/nihr.ac.uk/integrated-workforce-framework/home] Accessed 20 May 2019.

[44] Graham ID, Logan J, Harrison MB, Straus SE, Tetroe J, Caswell W, Robinson N (2006). Lost in knowledge translation: time for a map?. J Contin Educ Heal Prof.

[45] Rycroft-Malone J, Burton C, Wilkinson J, Harvey G, McCormack B, Baker R, Dopson S, Graham I, Staniszewska S, Thompson C, et al. Collective action for knowledge mobilisation: a realist evaluation of the Collaborations for Leadership in Applied Health Research and Care. Health Serv Delivery Res. 2015;3(44). 26677504

[46] Competency Framework for Clinical Research Nurses [https://matrix.rcn.org.uk/__data/assets/pdf_file/0019/201466/Research_Nurse_Competency_Framework_-_Version_2_-_Full_-_Oct_2011.pdf] Accessed 20 May 2019.

[47] Declaration of Helsinki–Ethical Principles for Medical Research Involving Human Subjects [https://www.wma.net/policies-post/wma-declaration-of-helsinki-ethical-principles-for-medical-research-involving-human-subjects/] Accessed 20 May 2019.

[48] Oliver D, Foot C, Humphries R, O'Neil K (2014). Making our health and care systems fit for an ageing population.

[49] Andrew MK, Rockwood K (2014). Making our health and care systems fit for an ageing population: considerations for Canada. Canadian Geriatr J.

[50] King O, Nancarrow SA, Borthwick AM, Grace S (2015). Contested professional role boundaries in health care: a systematic review of the literature. J Foot Ankle Res.

[51] Humphreys A, Johnson S, Richardson J, Stenhouse E, Watkins M (2007). A systematic review and meta-synthesis: evaluating the effectiveness of nurse, midwife/allied health professional consultants. J Clin Nurs.

[52] Health Services & Policy Research Enriched Core Competencies [http://www.cihr-irsc.gc.ca/e/49883.html] Accessed 20 May 2019.

[53] Capacity development framework [http://www.cihr-irsc.gc.ca/e/documents/spor_capacity_development_framework-en.pdf] Accessed 20 May 2019.

[54] Physiotherapy Framework: putting physiotherapy behaviours, values, knowledge & skills into practice [http://www.csp.org.uk/documents/physiotherapy-framework-condensed] Accessed 20 May 2019.

[55] NIHR CRN (2018). National Institute Health Research Clinical Research Network Allied Health Professionals Strategy 2018–2020.

[56] Vitae Researcher Development Framework [https://www.vitae.ac.uk/researchers-professional-development/about-the-vitae-researcher-development-framework] Accessed 20 May 2019.

[57] Clinical Academic Careers Pathway Capability Framework [file://ds.leeds.ac.uk/staff/staff4/hcsjharb/Downloads/AUKUH-Clinical-Academic-Careers-Capability-Framework-May-2014.pdf] Accessed 20 May 2019.

[58] National Job Profiles Allied Health Professionals: Generic Therapy [http://www.nhsemployers.org/-/media/Employers/Documents/Pay-and-reward/Generic_Therapy.pdf?la=en&hash=8A52DC70F739D3A51B0BAE06F68C28465E6491D4] Accessed 20 May 2019.

[59] Grafton K: RESearch Self-Assessment Tool (RESSAT) In: Sheffield Hallam Unibersity; 2017.70. Cambridge Dictionary [https://dictionary.cambridge.org/us/dictionary/english/stem] Accessed 20 May 2019.

[60] Advanced practice in physiotherapy: Understanding the contribution of advanced practice in physiotherapy to transforming lives, maximising independence and empowering populations [file://ds.leeds.ac.uk/staff/staff4/hcsjharb/Downloads/csp_advanced_practice_physiotherapy_2016_2.pdf] Accessed 20 May 2019.

[61] Dietitians and Research: A Knowledge and Skills Framework [https://www.bda.uk.com/professional/research/knowledgeskillsframework] Accessed 20 May 2019.

[62] Speech & Language Therapists working in Consultant Roles [https://www.rcslt.org/account/login?d=https%3A%2F%2Fwww.rcslt.org%2Fdocs%2Fconsultant_policy] Accessed 20 May 2019.

[63] Education and Career Framework for the Radiography Workforce [https://www.sor.org/learning/document-library/education-and-career-framework-radiography-workforce/14-researchers] Accessed 20 May 2019.

[64] Career Framework Guide: Prosthetics & Orthotics [https://www.bapo.com/Framework/ResourceManagement/GetResourceObject.aspx? ResourceID=71190978-13cb-4113-b230-6e50ad69fdba] Accessed 20 May 2019.

[65] Post Registration – Paramedic Career Framework [file://ds.leeds.ac.uk/staff/staff4/hcsjharb/Downloads/June_Final_Paramedic_Career_Framework_4th_edition_2018_-_for_website.pdf] Accessed 20 May 2019.

[66] Career Development Framework: Guiding Principles for Occupational Therapy 2017 [https://www.rcot.co.uk/practice-resources/learning-zone/career-development-framework] Accessed 20 May 2019.

[67] Canby A, McCrum C, Poole K (2017). East Sussex Research Escalator Tool. In*.*: East Sussex Healthcare NHS Trust, NHS England, Council for Allied Health Professions in Research.

