# Supplementary material for: Developing a consolidated research framework for clinical allied health professionals practising in the UK
Source: BMC Health Serv Res. 2020 Sep 11;20:852. doi: 10.1186/s12913-020-05650-3 (PMC7488424; doi:10.1186/s12913-020-05650-3)
Supplement: Supplementary file 1 — Additional file 1. Draft 1 Shaping Better Practice through Research: A Practitioner Framework. [file 12913_2020_5650_MOESM1_ESM.docx]

Additional file 1: Draft 1

Shaping Better Practice through Research: A Practitioner Framework

Principles of the Framework

1. The generation and application of research should be embedded in health and social care practice in order to improve services, promote health, wellbeing and safety of service users, and to optimise the effective use of resources.
2. All AHPs should enter their profession with research skills and knowledge at ‘Awareness’ level. This supports the notion of making research ‘core business’ to practice.
3. The framework can be used to plan the research element in health and social roles in a range of contexts.
4. Additional competencies unique to each professional may need to be developed to complement this consolidated framework, and to maximise their contribution to the research endeavour.
5. The framework portrays linear development but acknowledges that individuals, and the context within which they work will offer different opportunities for progress across different domains at different rates.
6. The framework should be used flexibly to plan workforce developments, profession career progression, and support systems needed to help embed applied research into organisational systems, for example through job descriptions, work plans, appraisal, mentorship and review systems.
7. This framework has been developed to meet the needs of AHPs, however it is not exclusive to them and may be relevant to other professional groups.
8. Research takes place in multi-professional and multi-disciplinary teams in a wide variety of health and social care settings. Many of the skills which support effective teamwork will also support research activity and are likely to maximise impact.

1. Research Methodology and Methods

| **A. Scientific concepts and application of research knowledge** | |
| --- | --- |
| Suggested entry Level | |
| Broad awareness of **knowledge creation processes** | Awareness |
| Awareness of basic **theoretical concepts** and **methodologies** in relation to applied research | Awareness |
| Understands **appropriate research methods to answer research questions** | Awareness |
| Critiques and selects appropriate **outcome measures / tools** in research projects | Awareness |
| Able to differentiate between **research, audit and service evaluation** | Awareness |
| Applies **technical language** with applied research  e.g.   - research participant compared to patient - data compare to information - statistical significance compared to clinical significance | Awareness |
| Contributes to **research questions** by considering research area and ‘real-world’ affairs | Core |
| **Application of theoretical concepts and methodologies** in relation to clinical research | Intermediate |
| Awareness of relevant research **methodological developments in field of interest** | Intermediate |
| **Uses multiple sources of evidence** (including stakeholder and user involvement / co-production) in research development | Intermediate |
| Articulates own **assumptions** and constructs and sustains arguments in a clear, evidenced and concise manner | Intermediate |
| **Work with stakeholders** throughout the research process | Intermediate |
| **B. Analysis** | |
| Suggested entry Level | |
| Is aware of appropriate tools and systems in the **search for evidence** e.g. databases | Awareness |
| **Information Technology** (IT) literate  For example, use of Excel, word | Awareness |
| Understands how to **interpret** qualitative and quantitative research data | Awareness |
| Undertakes appropriate **data analysis** | Core |
| Uses appropriate **tools** to collect data and measure outcomes | Core |
| **C. Proposal Development Level** | |
| Suggested entry Level | |
| Applies for **funding** grants and fellowships | Intermediate |
| **Designs research studies** using appropriate method s and methodologies for the research question | Intermediate |
| Writes research proposals that **adhere to requirements** of funding bodies, ethics and governance processes | Intermediate |
| Plans and leads detailed **research programmes** | Advanced |

2 Research Strategy and Planning

| **A. Applied research strategy and policy** | |
| --- | --- |
| Suggested entry Level | |
| Knowledge of **ethics** related to applied research | Core |
| Understands the value of **stakeholder involvement** (public, patient, and clinical involvement/engagement) in the selection of research priorities and throughout the applied research projects | Core |
| **Champions** the role of applied health and social care research to enhance health and wealth | Core |
| Has knowledge of advances in own and related research areas in order to recognise **gaps in research knowledge** | Intermediate |
| Knowledge of local and national **research policy** and its relevance to own practice area e.g NIHR, Scottish and welsh equivalent. | Intermediate |
| Contributes to local and national st**rategic vision** of research in health and social care settings | Intermediate |
| **B. Research Project Planning and Development** | |
| Suggested entry Level | |
| **Develops research questions relevant to policy and practice** | Awareness |
| Undertakes **critical thinking** to identify problems and research questions. | Awareness |
| Has knowledge of a range of study **designs and methodologies** relevant to applied research in relevant health and social care settings | Core |
| Understanding of different **phases of research process** | Core |
| Knowledge of the requirements for **Public and patient involvement/ engagement (PPIE)** in applied research | Core |
| Awareness of **regulatory and legal frameworks** and their implications for applied research design, and in the context range of health and social care settings | Intermediate |
| Understanding of **funding sources**. | Intermediate |
| Understanding of **financial management** in the design and conduct of research | Intermediate |
| Applies appropriate **budgeting practices** in research projects | Intermediate |
| Develops **research** proposals consistent with practice and policy **priorities** | Intermediate |

3 Research Delivery

| **Ethics, Safety and informed consent** | |
| --- | --- |
| Suggested entry Level | |
| Understands **confidentiality** regarding data and patient identifiable data including Caldecott principles | Awareness |
| Knowledge of **ethics and governance** approval procedures in relation when to start and deliver research | Awareness |
| Is able to undertake **consent and participant recruitment** in an ethical manner consistent with the research protocol (screening, randomization and data collection) | Awareness |
| Understands **adherence to protocols** and how this impacts on quality of the research | Core |
| Knowledge of **safeguarding** as part of developing research protocol | Core |
| Knowledge of **legal requirements** of research  e.g. data protection/ mental capacity/ Human tissue acts | Core |
| **Communicates complex information** in the context of conducting applied research | Core |
| Undertakes appropriate **risk assessment,** reflecting patient safety as part of developing and delivering research protocol | Core |
| Enables **Public and Patient Involvement /Experience (PPIE)** in the recruitment and delivery process | Core |
| Demonstrates **ethical performance in the planning of research delivery** (including equity of access, consent and opting out, consent for special groups, on-going consent and cultural variation) | Intermediate |
| **Knowledge of licensing authorities and** the licensing of investigational products, medical devices and IMP as applicable to role | Advanced |
| 1. **Operation of research** | |
| Suggested entry Level | |
| Has an understanding of different **communication strategies** and how they may be applied to different groups to maximize engagement in research | Awareness |
| Undertakes **Good Clinical Practice (GCP)** in relation to direct patient/participant care | Awareness |
| Is aware of professional responsibilities and **potential for conflict** with research role | Core |
| Delivers research activity as part of practice in line with **local procedures and national occupational standards** | Core |
| Undertakes **clinical care** **within the research project that is within their scope of practice**, or accesses training in order to do this | Core |

4 Research Management and Leadership

| 1. **Leadership and management in research** | |
| --- | --- |
| Suggested entry Level | |
| Has a knowledge of current **research leadership frameworks** and approaches (eg NIHR Leadership Framework, VITAE, NHS improvement themes etc) | Core |
| **Motivates, encourages and inspires others**, maintains own enthusiasm | Core |
| Identifies and **engages relevant stakeholders** in research projects | Intermediate |
| **Committee membership** related to research (research ethics committees, grant provider committees etc.) | Intermediate |
| Influences and leads less experienced researchers and **builds capability, creating a culture** of creativity and enquiry | Intermediate |
| **Creates ideas**, recognises good ideas and opportunities and acknowledges the contribution of others | Intermediate |
| **Identifies and responds to ethical and professional conflicts** | Intermediate |
| **Resilient** in the face of challenges, demonstrating self-reflection and striving to develop strengths and address weaknesses. | Intermediate |
| Awareness of the potential **research impact** on the professions and service provision | Intermediate |
| **Creates a nurturing and supportive culture** | Advanced |
| **Persuades and influences** a range of stakeholders to engage with research activity and evidence-based practice. | Advanced |
| Generates enthusiasm, presents and defends ideas that encourage people to **think differently** | Advanced |
| **Acts as a role model** and makes an identifiable contribution to evidence-based change & development within the profession or service & beyond | Advanced |
| 1. **Management and leadership in projects** | |
| Suggested entry Level | |
| **Research and development coordination** role on a site | Core |
| Understanding and knowledge of **principles of managemen**t of research project. | Core |
| Awareness of **roles within research project**. | Core |
| Knowledge of local **organisational and governance policies** e.g. Research & development processes and policies | Core |
| Awareness of funding constraints and **finance processes** | Core |
| Knowledge of **staff recruitment** processes | Intermediate |
| **Develops research teams** and infrastructure appropriate to the requirements of the research project | Intermediate |
| Develops effective **lines of communication** between different roles within research team (i.e. between sponsor, NHS Research Offices and research site) and within organisational structures | Intermediate |
| Manages **Intellectual Property (IP) and copyright** requirements | Intermediate |
| Effectively **manages time (self and others), budget and delivers project** component(s) on schedule | Intermediate |
| **Manages risk in** the conduct of research to maintain and improve research quality | Intermediate |
| **Performance management** of research team | Advanced |
| States clear expectations, clarify goals and negotiates realistic deadlines so that **people know what is expected of them** | Advanced |
| Implements procedures for **dealing with ethics and professional conflicts** | Advanced |
| **Plans and deals with unexpected changes** by maintaining a strategic view of project | Advanced |
| Undertakes **reporting requirements** related to research projects  Examples include: institutional review boards/independent ethics committees, sponsors, funders and regulatory authorities | Advanced |
| **Oversees** research across departments and services eg **multisite projects**. | Advanced |

5 Research Education and Training

| **A Education General (any setting)** | |
| --- | --- |
| Suggested entry Level | |
| Is aware of different **learning and teaching styles** and techniques | Awareness |
| Attends local **research and teaching events** eg conferences, journal clubs, study days | Awareness |
| Engages in **peer support, mentorship and supervision** of less-experienced researchers, to nurture talent and promote empowerment and autonomy | Core |
| **Educates colleagues in audit skills, service development and evidence-based practice** | Core |
| **Educates colleagues in research methodology, methods, and statistics** | Intermediate |
| Contributes to and promotes the **professional development** of the workforce in relation to research | Intermediate |
| **Showcases and uses data/findings** from research consultancy/ service development projects undertaken by staff, students or collaborators | Intermediate |
| Has a unique knowledge and **acts as an** **expert resource** for staff | Intermediate |
| **Acts as strategic link** between practice, professional bodies research institutes and academia to develop and influence research education provision. | Advanced |
| **Educates** specialist and non-specialist audiences **in complex ideas and theories.** | Advanced |
| 1. **Clinical Education** | |
| Suggested entry Level | |
| Ensure that pre-registration practice **education facilitates research informed approaches.** | Awareness |
| Knowledge of relevant **skills frameworks** eg professional body frameworks, vitae, HEE / NIHR etc | Core |
| 1. **Academic Education** | |
| Suggested entry Level | |
| Uses **research informed teaching** and learning methods which explicitly draw upon and use research in the discipline | Awareness |
| Develops **critical appraisal skills** in staff and students to understand ‘what is good research’ | Core |
| Is involved in **student assessment** and monitors and maintains the quality of assessment | Core |
| Undertakes **learning and development** **research** | Intermediate |
| Provides students and practitioners with opportunities and **experience of undertaking research** | Intermediate |
| **Devises research-informed learning** and development **programmes** including specialist research skills training | Intermediate |
| Supports students and practitioners through to **publication** and dissemination. | Intermediate |
| Knowledge of education requirements at pre-registration level to inform **curriculum development and planning** | Intermediate |

6 Working with Others and Collaborating in Research

| **A. Networking** | |
| --- | --- |
|  | Suggested entry Level |
| Knowledge of local **service-user involvement** strategies, groups and activities | Awareness |
| Awareness of **local and national research forums** related to clinical research | Awareness |
| **Networking and relationship building** to enhance research opportunities and activity  e.g. professional bodies, professional networks, CAHPR, NIHR infrastructure  Ambassadors, mentors | Awareness |
| Understands and recognises appropriate **stakeholders** (academic, professional and service users) to develop research proposals | Core |
| **Plans communication** between partners, stakeholders and research teams | Core |
| **Uses networks** to engage with colleagues and respond to opportunities | Core |
| Implements and adapts a range of **communication tools** **and channels** for the relevant audience | Core |
| Supports **service-user involvement networks** for research | Core |
| **Shares networks** with less experienced staff | Core |
| Establishes **networks across boundaries** e.g. health and social care systems and Higher Education Institutes | Intermediate |
| Attends and reports at **a senior level** (e.g. boards, executive committees) regarding **research-related governance, policy and service development** | Advanced |

7 Research-Informed Practice, Dissemination and Impact

| 1. **Translation of knowledge into practice** | |
| --- | --- |
| Suggested entry Level | |
| Uses **evidence-informed approaches** and a range of evidence sources including research, scholarship and continuing professional development to inform practice | Awareness |
| Uses **Critically appraised evidence** to address problems and issues arising in practice | Awareness |
| Critiques / **evaluates local practice** using a range of techniques including standardised tools / measures and innovative methodologies | Awareness |
| **Communicates** relevance of **research findings** and best practice to colleagues, advocacy groups and wider community | Core |
| Promotes **evidence-based practice** to improve service user outcome, patient experience and organisation culture | Core |
| Works with stakeholders, including patients and members of the public to **co-produce outputs that are usefu**l to them  e.g. check lists, training materials, decision aids, Patient Reported Outcome Measures | Intermediate |
| 1. **Dissemination of own research** | |
| Suggested entry Level | |
| Understands concept of **authorship and intellectual property** | Awareness |
| Understands the value of **open access publications** | Awareness |
| Familiar with processes **for peer review publication** | Awareness |
| Aware of local **outlets for dissemination** | Awareness |
| Develops **research outputs** that reflects the preferred **content and communication method of the target audience** | Core |
| Uses **interactive technologies** and has an online presence for research.  For example twitter, skype, webchats, webinar | Intermediate |
| **Contributes to peer review processes** | Intermediate |
| 1. **Impactful Activities** |  |
| Suggested entry Level | |
| Engages with **knowledge mobilisation practices**  For example in service training, Communities of Practice; service improvement methodologies (e.g. ‘plan: do: study: act’; micro systems) | Core |
| Knowledge of what **research impact is** | Core |
| Develops **research outputs aimed at benefiting practice or health outcomes** | Intermediate |

1. Career Development

| **Career development knowledge and skills** | |
| --- | --- |
| Suggested entry Level | |
| Aware of **own abilities and development needs** in both practice and academic fields | Awareness |
| Plans own **research development career pathways**  e.g. Integrated Clinical Academic pathways from NIHR ( internship> pre-doctoral> doctoral> clinical lecturer> Professor)  And  Staff nurse> Advanced Clinical Practitioner> Consultant | Awareness |
| **Discusses research career development with line manager** | Awareness |
| **Develops resilience and skills to deliver research** in demanding environments | Awareness |
| Knows who to contact to get **support with career planning** e.g. Mentorship, Research Design Service, manager through annual appraisal and other methods. | Awareness |
| **Acquires the relevant research credentials** | Core |
| Sets realistic and achievable **research career goals** | Core |
| Demonstrates **transferability of skills and experience** | Core |
| Takes advantage of broad range of **research employment and professional development opportunities** e.g. Training, secondment opportunities, taking on more divers roles within team (audit lead, recruitment of patients to portfolio projects) | Core |
| Consolidates and develops **specialist clinical skills** and expertise to **integrate with research skills.** | Core |

This research was funded and supported by CAPHR South Yorkshire www.cahpr.csp.org.ukand the NIHR CLAHRC Yorkshire and Humber (NIHR CLAHRC YH). [www.clahrc-yh.nihr.ac.uk](http://www.clahrc-yh.nihr.ac.uk). The views and opinions expressed are those of the author(s), and not necessarily those of the NIHR or the Department of Health and Social Care.
